# Mini‐Catalytically Inactive Cas13X‐Derived RNA Base Editing of β‐Catenin Attenuates Pulmonary Damage in a Murine Acute Lung Injury Model

**DOI:** 10.1002/mco2.70716

**Published:** 2026-03-30

**Authors:** Wenyi Liu, Wanda Bi, Saiying Hou, Juan Du, Ling Zeng, Anqiang Zhang, Huacai Zhang, Dalin Wen, Qingli Cai, Chu Gao, Ping Lin, Min Wu, Li Li, Jianxin Jiang

**Affiliations:** ^1^ Department of Trauma Medical Center,State Key Laboratory of Trauma and Chemical Poisoning Daping Hospital Army Medical University Chongqing China; ^2^ Biological Science Research Center Southwest University Chongqing China; ^3^ Wenzhou Institute University of Chinese Academy of Sciences Wenzhou China; ^4^ Department of Respiratory Medicine Daping Hospital Third Military Medical University (Army Medical University) Chongqing China

**Keywords:** *β*‐catenin, acute lung injury, alveolar type II epithelial cells, base editing, CRISPR‐Cas13

## Abstract

Acute lung injury (ALI) is characterized by a considerable mortality rate and currently lacks viable therapeutic strategies. Alveolar type II epithelial cells (AT2 cells) play a critical role in lung injury repair, potentially through activation of the Wnt/β‐catenin signaling cascade, which may enhance regenerative ability of lung tissue. In this study, we developed a mini‐catalytically inactive Cas13X (mini dCas13X)‐based adenosine‐to‐inosine (A‐to‐I) RNA editing approach, designated as β‐*ca*tenin T41 editing to t*r*ea*t* alv*e*o*l*ar type 2 cells (CARTEL), with the objective of alleviating lung damage in ALI. We found that CARTEL proficiently performed base editing on β‐catenin, achieving a high A‐to‐I conversion rate with minimal off‐target effects. Moreover, CARTEL significantly inhibited the degradation of β‐catenin, amplified Wnt/β‐catenin signaling activation and facilitated cellular proliferation. In a murine model of lipopolysaccharide (LPS)‐induced ALI, a single adeno‐associated virus (AAV)‐mediated administration of CARTEL effectively and primarily transduced AT2 cells, resulting in attenuated lung injury, enhanced AT2 cell proliferation, and improved pulmonary function, with no detected long‐term risks. Collectively, these findings revealed that CARTEL‐mediated RNA editing represents a promising therapeutic strategy to counteract lung injury occurring in diverse settings.

## Graphical abstract

AAV6.2FF‐delivered CARTEL (mini‐dCas13X‐mediated A‐to‐I editor) introduces c.121A>G editing in β‐catenin RNA of alveolar type II cells, leading to sustained Wnt/β‐catenin activation, robust AT2 proliferation, significant attenuation of LPS‐induced acute lung injury, and no excess off‐target edits or fibrotic toxicity for 4 weeks.

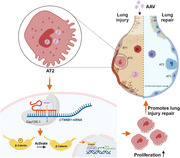

## 1 INTRODUCTION

The lung is particularly vulnerable among various organs in critical health conditions. Acute lung injury (ALI), particularly in its most severe manifestation known as acute respiratory distress syndrome (ARDS), is associated with high mortality and remains largely limited to supportive management, with few effective targeted therapies available [1, 2]. Diffuse lung epithelial injury is a major factor for the onset and progression of ALI/ARDS, leading to intractable hypoxemia and ultimately lung failure [3]. Accordingly, there is an urgent unmet need for therapeutic strategies that can actively promote alveolar repair and functional recovery.

Alveolar type II epithelial cells (AT2 cells) are pivotal progenitor cells involved in the repair and regeneration processes following lung injury [4]. Their regenerative capacity is tightly regulated by the canonical Wnt/β‐catenin signaling pathway, which sustains AT2 cell stemness and proliferative competence; conversely, inhibition of this pathway impairs AT2 cell expansion during lung repair [5, 6, 7]. Phosphorylation at T41 contributes to β‐catenin degradation; preventing phosphorylation stabilizes β‐catenin and thereby enhances Wnt/β‐catenin signaling [8]. Therefore, β‐catenin T41 may serve as a promising target for promoting the proliferation of AT2 cells.

The clustered regularly interspaced short‐palindromic repeat (CRISPR)‐Cas13 system serves as an RNA‐guided platform for programmable RNA targeting and editing, enabling post‐transcriptional manipulation of disease‐relevant transcripts without altering genomic DNA. This feature is particularly attractive for acute, safety‐critical indications, where a tunable and potentially reversible intervention is desirable and where precise control over signaling intensity may reduce risks associated with broad pathway modulation. In this context, RNA base editing has emerged as a powerful strategy to introduce defined nucleotide substitutions at the transcript level, thereby modulating protein function and downstream signaling outputs in a site‐specific manner [9].

Previously, cytidine deaminase was fused to dCas13b to develop a programmable RNA Editing for Specific cytidine‐to‐uridine (C‐to‐U) Exchange (RESCUE), which was effectively utilized to target and modify the β‐catenin transcript (*CTNNB1*) at the critical phosphorylation site T41 (changing ACC to AUC), achieving an editing rate of 28%, a fivefold increase in Wnt/β‐catenin signaling activation, and enhanced cell growth in HEK293T cells [8]. These results highlighted the feasibility of precisely rewiring Wnt/β‐catenin signaling through single‐site RNA editing but also underscored a practical limitation for in vivo translation: the substantial molecular weight of many Cas13 effectors poses obstacles for administration using a single adeno‐associated virus (AAV) vector. Recently, a streamlined RNA base editor [mini dCas13X‐based adenine base editor (mxABE)] has been engineered, which was composed of engineered deaminase (385 aa) and truncated Cas13X.1 (445 aa), exhibiting robust editing efficiency and high specificity for RNA base conversions both in vitro and in vivo via a single AAV vector [10]. This compact architecture provides a more realistic route for lung‐directed delivery and supports the therapeutic potential of Cas13X‐derived RNA editing for clinically relevant applications.

Here, we developed a targeted adenosine‐to‐inosine (A‐to‐I) RNA editing strategy, designated β‐*ca*tenin T41 editing to t*r*ea*t* alv*e*o*l*ar type 2 cells (CARTEL). CARTEL utilized A‐to‐I editing, which is read as A‐to‐G during translation and sequencing, to introduce the c.121A>G mutation in *CTNNB1* and thereby edit β‐catenin. This activated the Wnt/β‐catenin signaling pathway in both HEK293T and AT2 cells, and significantly alleviated lung injury in an LPS‐induced mouse model of ALI. Collectively, CARTEL presents a promising therapeutic avenue for the management of ALI.

## 2 RESULTS

### 2.1 CARTEL Efficiently Performed Base Editing of β‐Catenin in HEK293T Cell Lines

**FIGURE 1:**
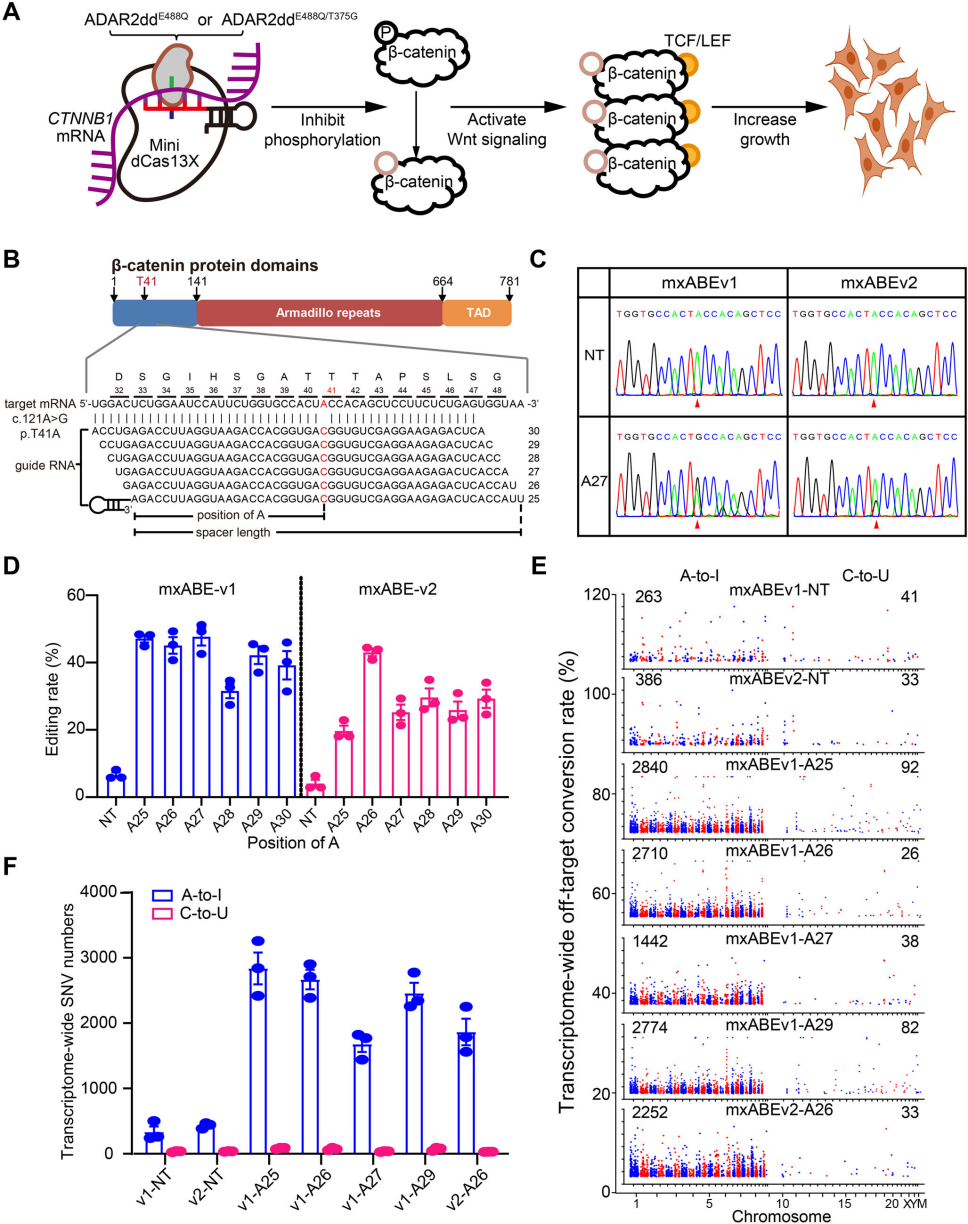
Efficient and precise base editing of CTNNB1 c.121A>G conversion in HEK293T cells. (A) Schematic of β‐catenin activation and cell growth utilizing mxABE‐mediated base editing. The p.T41A mutation in β‐catenin inhibits its phosphorylation and degradation, consequently, it activates the Wnt signaling pathway. Created with BioRender.com. (B) Schematic of β‐catenin domains and gRNAs of mxABE targeting the phosphorylation site at T41. (C) Sanger sequencing results for in vitro editing with base editors using different gRNAs, with red arrowheads indicating the target nucleotide. (D) The impact of mismatch position on A‐to‐G (I) conversion rate achieved by base editors with different gRNAs, as assessed by targeted deep sequencing. (E) Transcriptomic distribution of off‐target editing events induced by different base editors with or without specific spacers. (F) Total single‐nucleotide variants (SNVs) of A‐to‐I and C‐to‐U identified across the transcriptome as off‐target events for different base editors (*n* = 3). Data are presented as means ± SEM. NT, nontarget gRNA. mxABEv1, mini dCas13X‐based adenine base editor version 1. mxABEv2, mini dCas13X‐based adenine base editor version 2.

To pinpoint optimal guide RNA, two mini A‐to‐I base editors, mxABEv1 (a fusing of mini dCas13X and ADAR2ddE488Q) and mxABEv2 (a fusing of mini dCas13X and ADAR2ddE488Q/T375G), which exhibited distinct editing selectivity and efficiency, were engineered to activate Wnt/β‐catenin pathway by editing key phosphorylation site T41 on β‐catenin transcript (*CTNNB1*). The c.121A>G conversion in *CTNNB1* results in the p.T41A mutation in β‐catenin, which blocks phosphorylation at threonine 41, thereby preventing its degradation. Stabilized β‐catenin accumulates in the nucleus, activates the Wnt signaling pathway, and promotes cell proliferation to accelerate tissue repair (Figure 1A).

A total of six 50‐nucleotide (nt) spacers, each incorporating a single nucleotide mismatch at positions 25–30 nt of *CTNNB1* 121A, were created (Figure 1B). The mxABEv1/v2 constructs with distinct spacers were transfected into human embryonic kidney (HEK) 293T cells, and GFP‐positive (GFP^+^) cells were sorted using flow cytometry for Sanger sequencing or targeted deep sequencing to evaluate the base editing activity. Sanger sequencing revealed variable editing efficiencies among the six gRNAs, with the highest A‐to‐G (I) conversion rate at position A27 for mxABEv1 (Figure 1C and Figure S1). Deep sequencing confirmed that mxABEv1‐A27 achieved the most efficient editing, with an A‐to‐G (I) conversion rate of 47.68 ± 2.62% (Figure 1D). To assess off‐target activity, we performed transcriptome‐wide RNA sequencing (RNA‐seq) of base editors with editing efficiency exceeding 40% (mxABEv1‐A25, ‐A26, ‐A27, ‐A29, and mxABEv2‐A26). Among the tested editors, mxABEv1‐A27 displayed the fewest off‐target A‐to‐G (I) editing events (1678.3 ± 118.9, Figure 1E,F). Therefore, based on its superior editing efficiency and minimal off‐target effects, mxABEv1‐A27 was selected for all subsequent experiments and hereafter referred to as CARTEL (T41A).

### 2.2 CARTEL Augmented Activation of Wnt/β‐Catenin Signaling Pathway

**FIGURE 2:**
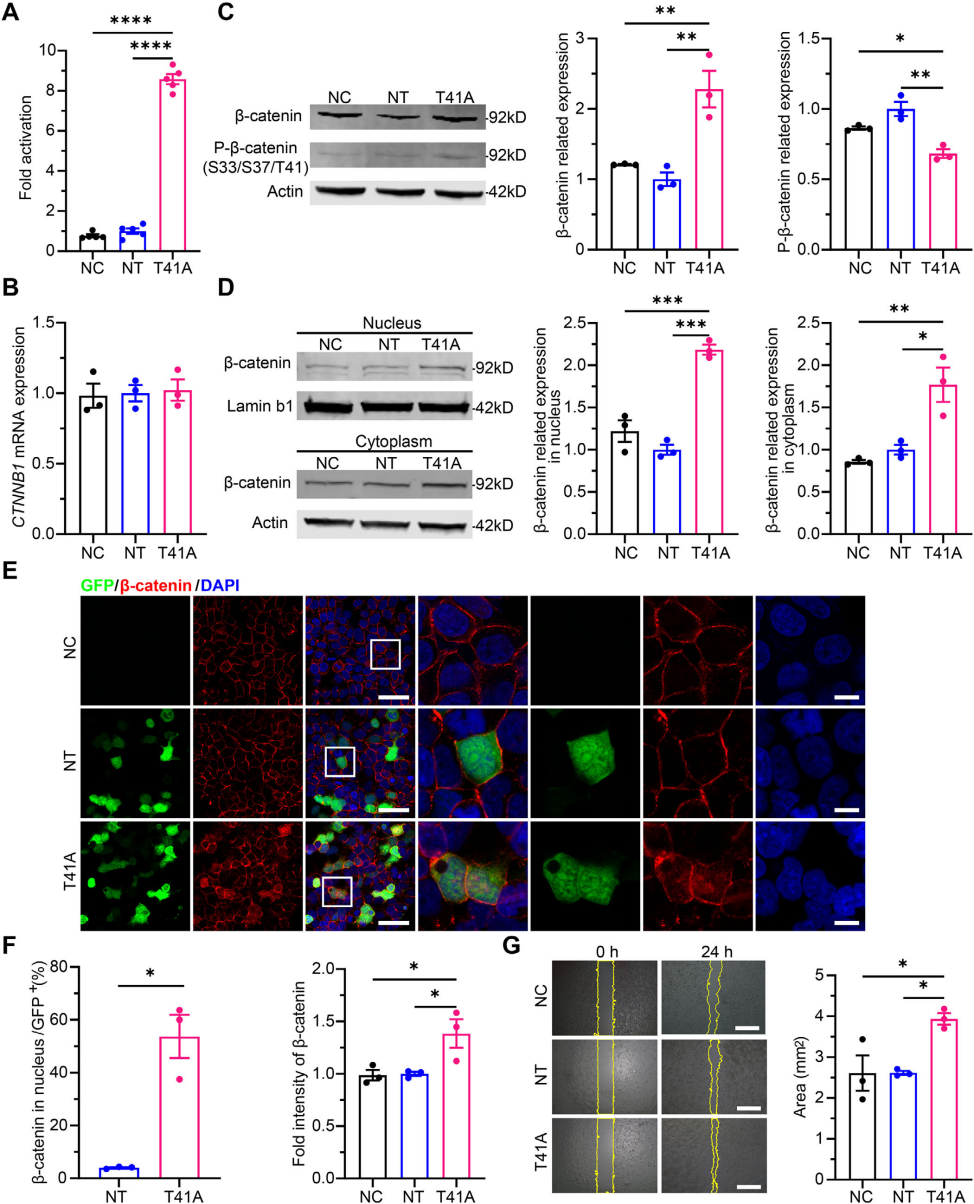
CARTEL activates Wnt/β‐catenin signaling and promotes cell growth in vitro. (A) Luciferase reporter assay showing increased Wnt/β‐catenin activity in CARTEL (T41A)–treated HEK293T cells compared with NT and negative control NC groups (*n* = 5). (B) qPCR analysis of CTNNB1 mRNA expression showing no significant difference among groups (*n* = 3). (C, D) Immunoblot analysis of total and phosphorylated‐β‐catenin expression in whole cell lysates, and in nucleus or cytoplasm of HEK293T cells transfected with CARTEL or controls. β‐Actin and lamin b1 served as loading control. Protein bands were normalized to β‐actin or lamin b1 and expressed as fold change versus NT (set to 1, *n* = 3). (E, F) Immunofluorescence staining of β‐catenin (red) and mxABE expressed GFP (green) in HEK293T cells, with DAPI (blue) marking nuclei. Quantification of cells with β‐catenin in the nucleus in total GFP‐positive cells (*n* = 3) and fold intensity of β‐catenin (*n* = 3). Scale bar, 50 µm (overview), 20 µm (magnified view). (G) Representative microscopy images and quantification of cellular growth due to the treatment of CARTEL in HEK293T cells (*n* = 3). Scale bar = 200 µm. Data are presented as mean ± SEM. *p < 0.05; **p < 0.01; ***p < 0.001; ****p < 0.0001. NC, negative control. NT, nontarget gRNA.

We next examined whether CARTEL could impact Wnt/β‐catenin signaling. Luciferase reporter assay revealed that CARTEL (T41A) increased signaling activity by 8.6 ± 0.3‐fold compared with the nontargeting guide (NT) group and markedly exceeded the negative control (NC) group (Figure 2A). Notably, qPCR analysis showed no change in *CTNNB1* mRNA levels (Figure 2B), indicating that CARTEL activates Wnt/β‐catenin signaling at the post‐transcriptional level. CARTEL significantly increased total β‐catenin levels in HEK293T cells, and quantification of the ratio between phospho‐ and total β‐catenin demonstrated a concomitant reduction in phosphorylated β‐catenin (Figure 2C). We further investigated the subcellular distribution of β‐catenin and found that CARTEL increased nuclear β‐catenin levels by 2.2 ± 0.1‐fold and cytoplasmic levels by 1.8 ± 0.2‐fold compared to the NT group (Figure 2D). Next, immunofluorescence staining results showed that significantly higher level of β‐catenin was seen in transfected cells in CARTEL group, especially in the nucleus (Figure 2E,F) compared to controls. Then, we examined whether CARTEL may enhance cell growth by quantifying the cell‐occupying area in HEK293T cells and found that CARTEL significantly increased cell growth at 24 h after transfection (Figure 2G). Taken together, these results indicate that CARTEL may inhibit the degradation of β‐catenin, promoting its increased expression in the nucleus and activating Wnt/β‐catenin signaling to promote cell growth in vitro.

### 2.3 AAV6.2FF Transduced Alveolar Type 2 Cells In Vivo

Efficient and cell‐selective gene transfer to AT2 cells remains challenging. The most effective and commonly used gene delivery vectors are those based on AAV [11]. AAV6 is highly effective in transducing airway epithelial cells [12], and an engineered AAV6 capsid, containing an amino acid substitution (F129L) [13], and two mutations (Y445F and Y731F), designated AAV6.2FF, has been reported to efficiently target AT2 cells [14].

**FIGURE 3:**
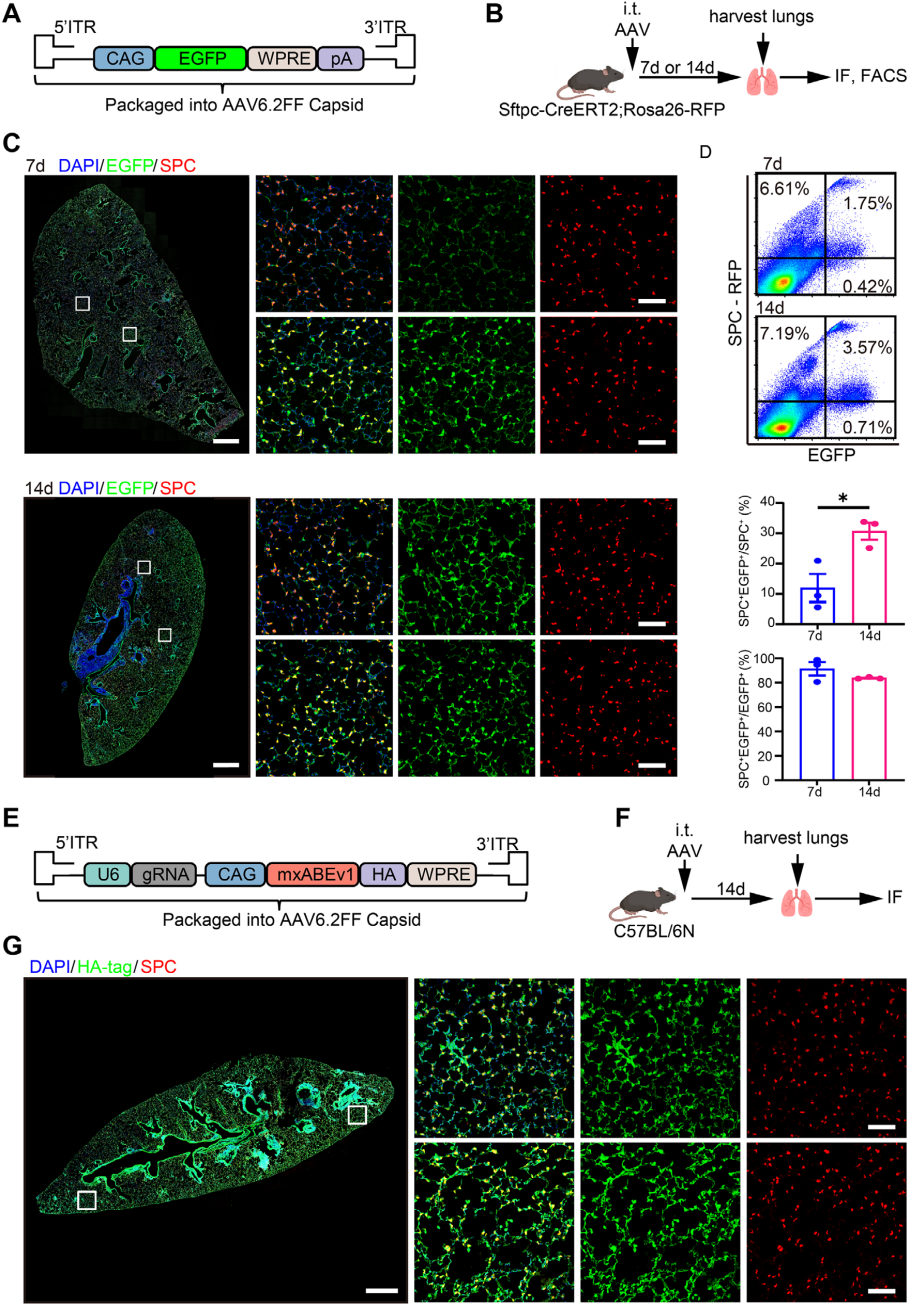
AAV6.2FF efficiently and selectively transduces AT2 cells in vivo. (A) Genome map of the EGFP‐reporter vector: CAG‐EGFP‐WPRE‐SV40pA flanked by AAV ITRs and packaged into the AT2‐tropic AAV6.2FF capsid. (B) Study design to determine the extent of alveolar type II cells transduction 7 or 14 days following intratracheal (i.t.) instillation of 1011 v.g. of AAV6.2FF‐EGFP into 8‐week‐old Sftpc‐CreERT2 and Rosa26‐RFP mice. (C) Representative immunofluorescence images of EGFP (green), Sftpc (red), and DAPI (blue) of mouse lung sections 7 or 14 days following AAV6.2FF i.t. instillation. Scale bars, left =1 mm, Right = 50 µm. (D) Representative flow cytometry images to detect GFP+ and Sftpc+ cells, and the quantitative result in mouse lung tissue at 7 and 14 days after AAV6.2FF instillation (*n* = 3). (E) Schematic of the AAV6.2FF vector carrying mxABEv1 for targeted editing of β‐catenin p.T41A (AAV‐T41A) or a non‐targeting control (AAV‐NT). (F) Experimental design for intratracheal delivery of 1 × 10^1^ ^1^ v.g. AAV‐T41A or AAV‐NT into C57BL/6N mice, with lung samples collected 14 days post‐injection. (G) Immunofluorescence images of mouse lung sections showing AAV‐transduced (HA^+^, green) cells, AT2 cells (Sftpc^+^, red), and nuclei (DAPI+, blue) 14 days following CARTEL (AAV‐T41A) intratracheal instillation. Scale bar, left = 1 mm, right = 50 µm. Data are presented as means ± SEM. *p < 0.05.

We then chose AAV6.2FF as the delivery vector and assessed its in vivo targeting ability in AT2 cells by intratracheally delivering 10^11^ vector genomes (v.g.) to Sftpc‐CreERT2 and Rosa26‐RFP mice (Figure 3A,B). Immunofluorescence staining showed that 7 days post‐delivery, Sftpc^+^ cells, typically considered AT2 cells, exhibited a homogeneous distribution throughout the lung, while EGFP^+^ cells were primarily localized near the large airways (Figure 3C, upper panel). After 14 days of AAV delivery, EGFP^+^ cells displayed a broader distribution and appeared to co‐localize with Sftpc^+^ AT2 cells (Figure 3C, lower panel). Meanwhile, flow cytometry analysis showed that 30.7 ± 2.8% of AT2 cells were EGFP^+^ at 14 days post‐injection, significantly higher than that of 12.0 ± 4.6% at 7 days (Figure 3D). On the other hand, the percentage of Sftpc^+^ cells among EGFP^+^ cells was similar between the two groups (91.4 ± 5.5% at 7 days and 83.8 ± 0.4% at 14 days, Figure 3D), suggesting that AAV6.2FF could effectively and selectively target AT2 cells especially at 14 days after delivery.

To evaluate the suitability of AAV6.2FF as a vector for delivering the RNA editing system to AT2 cells, 10^11^ v.g. per mouse of AAV6.2FF carrying mxABE plasmid for β‐catenin p.T41A (CARTEL, hereafter referred to as AAV‐T41A) or a non‐targeting control (AAV‐NT) was intratracheally administered to C57BL/6N mice (Figure 3E,F). After 14 days, AAV‐transduced cells (HA^+^) were uniformly distributed throughout lung sections and predominantly co‐localizing with AT2 cells (Sftpc^+^) (Figure 3G and Figure S2). For safety considerations, compared to PBS‐treated mice, delivery of AAV6.2FF vector containing EGFP (AAV‐EGFP), mxABEv1‐NT (AAV‐NT), or mxABEv1‐T41A (AAV‐T41A) had no impact on body weight (Figure S3), and no evidence of hemorrhage or macroscopic lung injury was observed, indicating that AAVs did not cause any apparent lung injury (Figure S4). Overall, these findings demonstrate that AAV6.2FF efficiently transduces AT2 cells to deliver CARTEL without causing apparent harm to the mouse.

### 2.4 CARTEL Increased β‐Catenin Expression and Activated Wnt Signaling Pathway in Vivo

**FIGURE 4:**
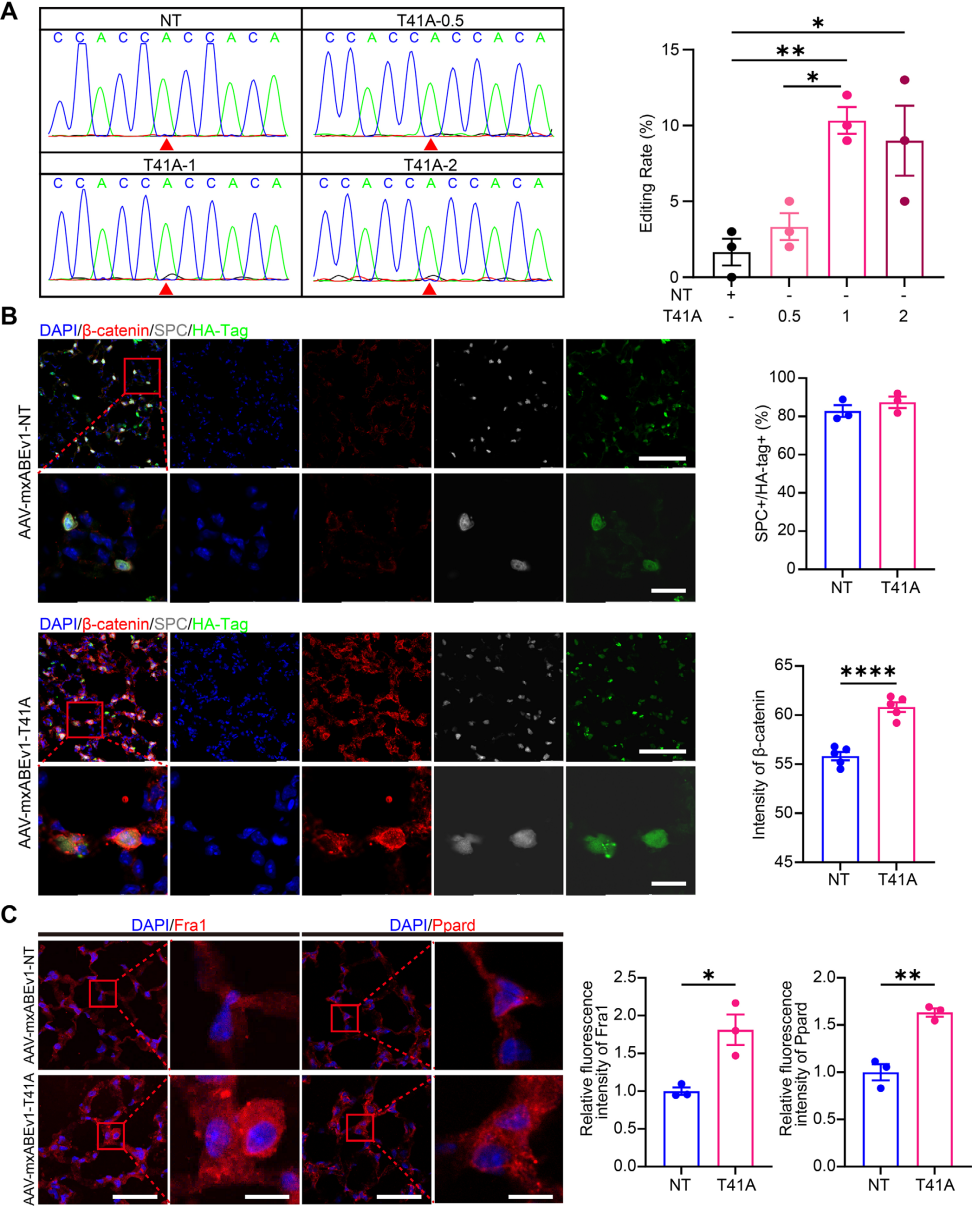
CARTEL editing of CTNNB1 increases β‐catenin expression and activates Wnt signaling in vivo. (A) Sanger sequencing and quantification of CTNNB1 c.121A>G editing in HA+ lung cells isolated from C57BL/6N mice 14 days after intratracheal injection of AAV‐mxABEv1‐NT and AAV‐mxABEv1‐T41A (CARTEL) at indicated doses (0.5 × 10^1^ ^1^, 1.0 × 10^1^ ^1^, or 2.0 × 10^1^ ^1^ v.g.). Red arrowheads indicate the target nucleotide (*n* = 3). (B) Immunofluorescence staining of lung sections from Rosa26‐RFP mice 14 days after AAV intratracheal instillation, showing AAV‐transduced (HA^+^, green) cells, AT2 cells (Sftpc^+^, red), and nuclei (DAPI+, blue). Quantification of Sftpc+HA+ cells among total HA+ cells (*n* = 3) and mean β‐catenin fluorescence intensity (*n* = 6). Scale bar, upper = 100 µm, lower = 20 µm. (C) Immunofluorescence staining of Fra1 (red) and Ppard (red) in lung sections from AAV‐mxABEv1‐NT and AAV‐mxABEv1‐T41A (CARTEL) groups, with DAPI (blue) marking nuclei. Quantification of fluorescence intensity is shown (*n* = 3). Scale bar = 50 µm. Data are presented as means ± SEM. *p < 0.05; **p < 0.01.

To investigate the dose–response correlation between AAV and editing rate, mice were intratracheally administered AAV‐CARTEL (T41A) at doses of 0.5 × 10^1^ ^1^, 1.0 × 10^1^ ^1^, or 2.0 × 10^1^ ^1^ v.g. On day 14, HA^+^ lung cells were isolated via FACS, and the editing of *CTNNB1 c.121*A>G (I) was quantified by Sanger sequencing. Editing rates for the 1.0 × 10^1^ ^1^ and 2.0 × 10^1^ ^1^ v.g. doses were significantly higher than NT group, while the 0.5 × 10^1^ ^1^ v.g. dose showed no significant difference from the NT group. Additionally, no significant difference was detected between the 1.0 × 10^1^ ^1^ v.g. and 2.0 × 10^1^ ^1^ v.g. dose groups (Figure 4A), with the 1.0 × 10^1^ ^1^ v.g. group achieving an editing rate of 10.3 ± 0.9%, which is consistent with editing efficiencies reported for mini dCas13X‐based systems [15]. Given the absence of additional benefit at the higher dose and to minimize vector load, 1.0 × 10^1^ ^1^ v.g. was adopted for all subsequent in vivo experiments.

Next, immunofluorescence staining of lung sections from Rosa26‐RFP mice showed markedly elevated β‐catenin signal in Sftpc^+^HA^+^ AT2 cells in AAV‐T41A (CARTEL) group compared to the control. Quantitative analysis confirmed that, with comparable proportions of Sftpc^+^HA^+^ cells among HA^+^ cells between groups (87.4 ± 3.0% in T41A group and 82.8 ± 3.1% in NT group), the mean β‐catenin fluorescence intensity in the AAV‐T41A group was significantly higher than that in the AAV‐NT group (60.8 ± 0.5 vs. 55.8 ± 0.4, Figure 4B).

To determine whether the increased β‐catenin expression led to functional activation of the Wnt signaling pathway and to explore the potential mechanism underlying AT2 cell proliferation, we examined the expression of two canonical Wnt target genes, *Ppard* and *Fra1*, which are critically involved in cell proliferation. Immunofluorescence staining of lung tissue sections revealed that Fra1 and Ppard protein levels were increased by 1.81 ± 0.20‐fold and 1.63 ± 0.04‐fold, respectively, in AAV‐T41A (CARTEL) lungs versus AAV‐NT controls (Figure 4C).

Taken together, these results suggest that the administration of CARTEL utilizing AAV6.2FF delivery facilitates RNA editing of the *CTNNB1* transcript (c.121A>G) in mouse lungs at a dosage of 1.0 × 10^11^ v.g., leading to increased β‐catenin expression and subsequent activation of its proliferative downstream effectors Ppard and Fra1.

### 2.5 CARTEL Promoted Mice Lung Repair After LPS Challenge

**FIGURE 5:**
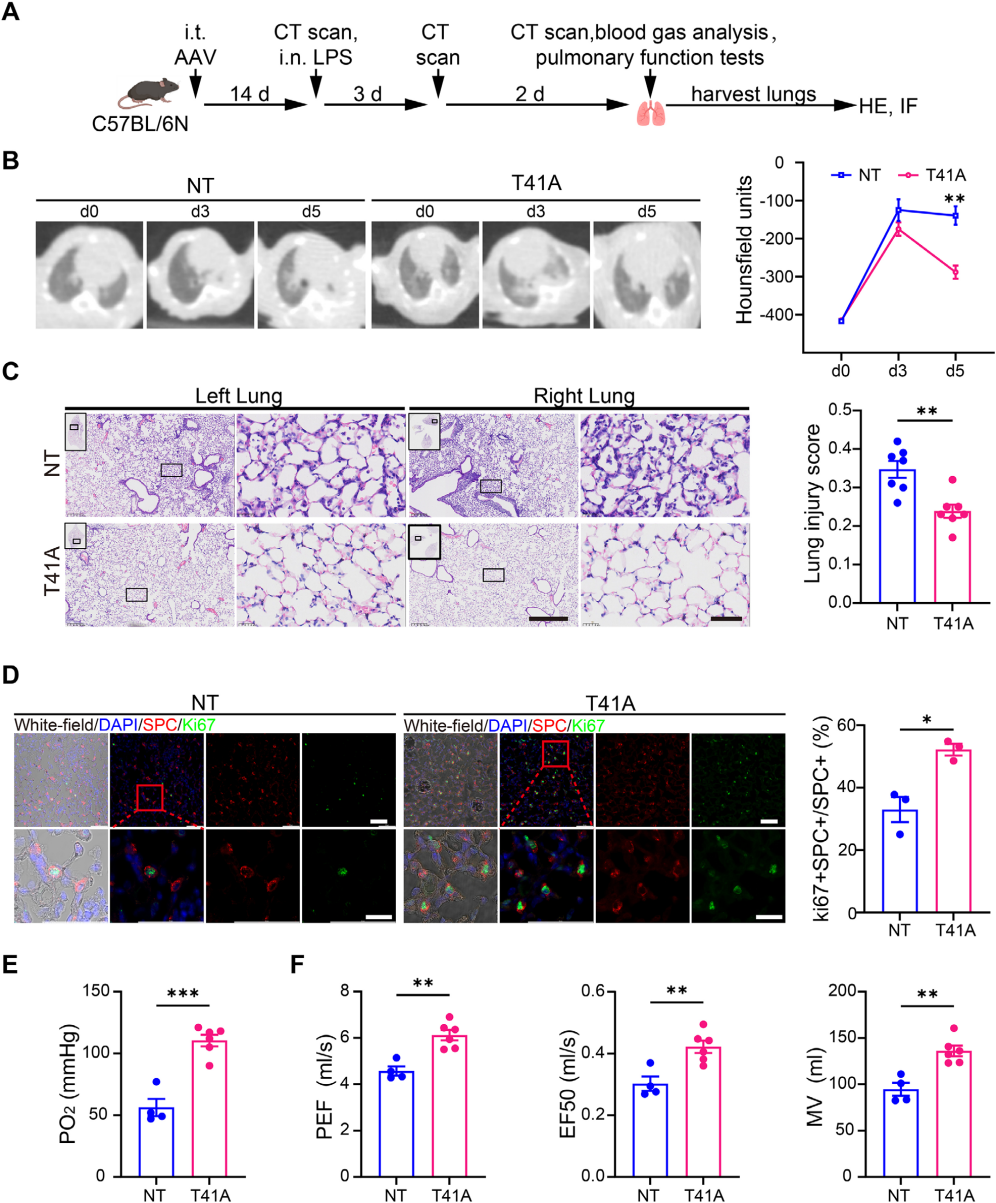
CARTEL promotes structural and functional lung repair following LPS challenge. (A) Study design. After 1 × 10^1^ ^1^ v.g. AAV6.2FF‐mxABEv1‐NT (NT) or CARTEL (AAV6.2FF‐mxABEv1‐T41A, T41A) was intratracheally delivered for 14 days, mice were treated with LPS (10 mg/kg) intranasally and observed for 5 days. (B) Representative micro‐CT images of mice from two groups with different AAV treatment as indicated, with quantification of hounsfield units (NT: *n* = 6, T41A: *n* =8). (C) Representative H&E staining of lung sections collected on day 5 post‐LPS, with quantification of lung injury scores (NT: *n* = 7, T41A: *n* = 7). Scale bars: overview = 500 µm; magnified view = 50 µm. (D) Immunofluorescence staining of Ki67 (green), Sftpc (white), and DAPI (blue) in mouse lung sections on day 5 post‐LPS. (Scale bar, upper = 50 µm, lower = 20 µm). Quantification of proliferating AT2 cells (Ki67^+^Sftpc^+^ among Sftpc^+^ cells) is shown (*n* = 3). Scale bars: overview = 50 µm; magnified view = 20 µm. (E) Arterial blood gas analysis showing PaO_2_ levels on day 5 post‐LPS (NT: *n* = 4, T41A: *n* = 6). (F) Pulmonary function measurements, including peak expiratory flow (PEF), expiratory flow at 50% of tidal volume (EF50), and minute ventilation (MV), on day 5 post‐LPS (NT: *n* = 4, T41A: *n* = 6). Data are presented as means ± SEM. *p < 0.05, **p < 0.01, ***p < 0.001.

To investigate the ability of CARTEL in promoting pulmonary repair in vivo, we established a lung injury model in C57BL/6N mice via intranasal instillation of lipopolysaccharide (LPS), which had received intratracheal delivery of AAVs for 14 days (Figure 5A). On day 3 post‐injury, both experimental groups exhibited comparable tissue damage, as evaluated by micro‐computed tomography, whereas the AAV‐T41A group displayed significant and rapid recovery of lung integrity by day 5 (Figure 5B). Additionally, the AAV‐T41A (CARTEL) group displayed lower lung injury scores compared to the AAV‐NT group on day 5 (0.24 ± 0.02 vs. 0.35± 0.02, Figure 5C). Moreover, multi‐color immunofluorescence staining showed a higher proportion of Ki67‐positive AT2 cells in the AAV‐T41A group (52.2 ± 1.9% vs. 33.0 ± 4.0%, Figure 5D).

To determine if the observed histological repair translated to improved respiratory function, we performed arterial blood gas analysis and pulmonary function tests on day 5 post‐LPS administration. Consistent with the structural recovery, CARTEL‐treated mice exhibited a remarkable improvement in gas exchange, as evidenced by a significantly higher arterial partial pressure of oxygen (PaO_2_) compared to the NT group (110.5 ± 4.7 vs. 56.0 ± 6.7 mmHg, Figure 5E). Furthermore, key parameters of pulmonary function, including peak expiratory flow (PEF), expiratory flow at 50% of tidal volume (EF50), and minute ventilation (MV), were also significantly improved in the AAV‐T41A (CARTEL) group (Figure 5F), indicating enhanced lung function. Collectively, these results suggest that CARTEL, delivered by AAV6.2FF, enhances both structural and functional pulmonary repair following LPS exposure through the stimulation of AT2 cell proliferation.

### 2.6 In Vivo Off‐Target Evaluation and Long‐Term Safety of CARTEL

**FIGURE 6:**
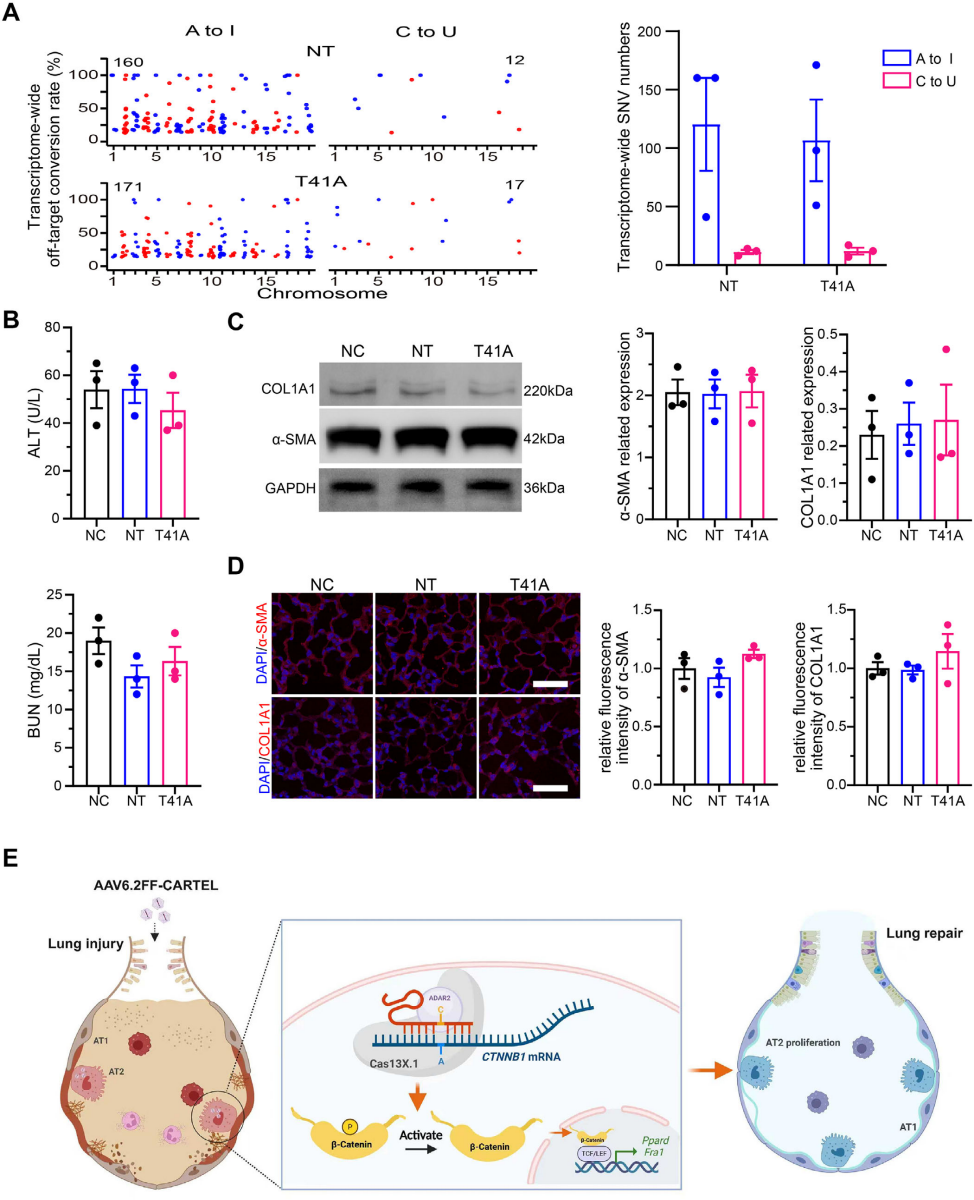
CARTEL does not increase off‐target editing or long‐term toxicity in vivo. (A) Transcriptome‐wide off‐target analysis. HA^+^ lung cells were FACS‐isolated 14 days after 1 × 10^1^ ^1^ v.g. AAV6.2FF‐T41A (CARTEL) or AAV6.2FF‐NT instillation and subjected to RNA‐seq. Total single‐nucleotide variants (SNVs) of A‐to‐I and C‐to‐U identified across the transcriptome as off‐target events for different groups (*n* = 3). (B) ALT and BUN levels 28 days post‐instillation of PBS (NC), AAV6.2FF‐NT (NT) or AAV6.2FF‐CARTEL (T41A) (*n* = 3). (C) Fibrosis evaluation by western blot. Representative images and quantification of COL1A1 and α‐SMA in whole‐lung lysates 28 days after treatment; GAPDH serves as loading control (*n* = 3). (D) Immunofluorescence images of lung sections stained for COL1A1 (red) and α‐SMA (red) 28 days post‐instillation; DAPI (blue) marks nuclei. Scale bar, 50 µm. (E) Schematic summary. Created with BioRender.com. AAV6.2FF‐CARTEL achieves efficient CTNNB1 c.121A>G editing in AT2 cells, prevents T41 phosphorylation of β‐catenin, thereby stabilizing the protein, amplifying Wnt/β‐catenin–TCF/LEF signaling, and up‐regulating downstream targets Ppard and Fra1. Consequently, AT2 cells proliferate and accelerate lung repair after LPS‐induced injury without excess off‐target events or long‐term toxicity. Data are presented as means ± SEM.

To investigate in vivo off‐target effects, mice were intratracheally administered AAV‐CARTEL (T41A) and AAV‐NT at dose of 1.0 × 10^1^ ^1^ v.g. On day 14, HA^+^ lung cells were sorted and RNA‐seq was performed. The results showed comparable in vivo off‐target events between CARTEL (T41A) group and NT group, including both A‐to‐I (T41A: 106.7 ± 34.9 vs. NT: 120.3 ± 40.0) and C‐to‐U sites (T41A: 12.0 ± 2.89 vs. NT: 11.3 ± 1.8) (Figure 6A). These findings indicate that CARTEL does not increase the transcriptome‐wide off‐target burden in vivo.

To further evaluate the long‐term safety of CARTEL, we extended the observation period to 4 weeks. Serum biochemistry analysis revealed no significant elevation in ALT or BUN levels in either the CARTEL (T41A) group or NT group, compared to saline‐treated mice (Figure 6B), indicating no liver or kidney dysfunction. Additionally, western blot analysis and immunofluorescence staining showed no significant differences in the levels of fibrosis markers, α‐SMA and COL1A1, between the CARTEL (T41A) and control groups (Figure 6C,D), suggesting that CARTEL treatment did not induce fibrosis.

Collectively, in a murine model of LPS‐induced ALI, a single AAV6.2FF‐mediated delivery of CARTEL effectively and primarily transduced AT2 cells, introducing the c.121A>G mutation in *CTNNB1*, which inhibited phosphorylation at the threonine 41 (T41) residue of β‐catenin. This led to suppressed β‐catenin degradation, amplified Wnt/β‐catenin signaling, and facilitated cellular proliferation, resulting in attenuated lung damage, enhanced AT2 cell proliferation, and improved pulmonary function, with no detected long‐term risks (Figure 6E). CARTEL presents a promising therapeutic avenue for the management of ALI.

## 3 DISCUSSION

ALI remains a major clinical challenge due to its rapid progression and lack of effective targeted therapies. Here, we show that the A‐to‐I RNA editing strategy, termed CARTEL, efficiently targeted β‐catenin T41 phosphorylation and augmented Wnt/β‐catenin signaling in vitro and in vivo. Moreover, by intratracheal administration of CARTEL via AAV6.2FF, we demonstrated its robust therapeutic efficacy and safety in a murine model of LPS‐induced ALI.

The canonical Wnt/β‐catenin pathway plays a critical role in regulating stem/progenitor cell activity in various organs, such as endothelial, epithelial, and stromal tissues [16]. Various strategies have been developed to modulate Wnt signaling. For example, engineered Fzd‐Lrp agonist (Fzd^ag^) antibodies can effectively activate Wnt signaling, stimulating AT2 cell proliferation without exacerbating fibrosis in both mice and humans [17]. Another study has shown that a specific Wnt mimetic can reduce pulmonary fibrosis and inflammation, thereby improving lung function in a bleomycin‐induced lung injury model [18]. While targeting Wnt signaling shows therapeutic potential for degenerative diseases, broad or non‐specific activation may have unintended consequences on different stem cell populations, potentially leading to serious side effects [19]. Therefore, it is crucial to develop strategies to specifically target and precisely regulate Wnt signaling pathways to overcome these potential challenges and side effects.

RNA editing technology represents a crucial platform for realizing tissue repair and regeneration in vivo. Xiao et al. demonstrated RNA correction therapy with Cas13‐based RNA base editors in the Myo6^C442Y/+^ mouse model, which mimicked dominant‐inherited deafness [15]. Injection of mxABE into the cochlea of neonatal *Myo6* ^C442Y/+^ mice improved auditory function by inducing A‐to‐G conversion in *Myo6* ^C442Y^ RNA. Additionally, systemic or local administration of mxABE significantly enhanced muscle development and functionality by targeting a nonsense point mutation in a genetically humanized mouse model of Duchenne muscular dystrophy [20].

Unlike upstream interventions such as CasRx‐mediated knockdown of Axin1/2 [21], which can trigger broad network effects, CARTEL specifically targets *CTNNB1* T41, directly linking the edit to downstream Wnt signaling outputs. In addition, the AAV6.2FF vector provides preferential AT2 cell tropism, enhancing delivery specificity. Although the in vivo A‐to‐I conversion in AT2 cells was modest (10.3 ± 0.9%), this level is comparable to prior in vivo reports of AAV‐delivered mini‐dCas13X editors [15], and further results demonstrated its therapeutic effect. CARTEL treatment resulted in significant histological and functional improvements in the LPS‐induced ALI model. Moreover, RNA‐seq detected no excess off‐target editing, and 4‐week serum and histology analyses revealed no liver, kidney, or fibrotic toxicity, confirming the biosafety of CARTEL. Collectively, CARTEL, a novel paradigm for in vivo RNA editing‐based regenerative therapy targeting post‐transcriptional regulation of critical signaling nodes in lung progenitor cells, not only provides a proof‐of‐concept for RNA base editing in ALI but also offers a potentially broadly applicable platform for other conditions involving β‐catenin dysregulation.

Importantly, CARTEL also has translational appeal as a lung‐regenerative intervention. The compact mini‐dCas13X editor enables single‐vector packaging for pulmonary delivery, and RNA base editing offers a reversible and titratable modality compared with permanent DNA editing. Nevertheless, several translational questions remain, including whether human AT2 cells can be efficiently and selectively targeted in the context of inflamed, protein‐rich airspaces, how to manage pre‐existing or induced immunity to AAV capsids and bacterial‐derived Cas proteins, and how to define pharmacodynamic benchmarks (editing level–response relationships) and durability requirements for clinical benefit. Finally, leveraging human‐relevant systems (e.g., lung organoids, precision‐cut lung slices, or ex vivo perfused lung tissue), together with scalable GMP manufacturing and standardized potency assays, will be essential to optimize the clinical translation of CARTEL.

However, several limitations should be noted. First, while editing efficiency in vivo is consistent with existing dCas13 systems, further optimization is needed to enhance efficacy without increasing off‐target risks. Second, the long‐term persistence of therapeutic effects beyond 4 weeks remains to be systematically evaluated. Third, while we did not include in vitro validation with primary AT2 cells, future work will incorporate these models to strengthen the translational relevance of CARTEL. Additionally, because we did not perform Wnt/β‐catenin blockade (e.g., IWP‐2 or XAV939), the causal requirement of Wnt/β‐catenin activation for CARTEL‐driven AT2 proliferation and lung repair remains to be established. Finally, although the current model addresses acute injury, the applicability of CARTEL to chronic or heterogeneous lung diseases needs further investigation. Addressing these limitations will be important priorities for future studies to further optimize CARTEL and advance its translational potential.

In summary, we found that mxABE‐mediated RNA editing technology, CARTEL, represents a safe, efficient, and highly specific RNA editing strategy for enhancing endogenous repair mechanisms via β‐catenin pathway activation in AT2 cells. Our findings substantiate the concept of RNA base editing as a viable therapeutic strategy for the management of lung injuries and other diseases.

## 4 MATERIALS AND METHODS

### 4.1 Plasmid Construction

Plasmids, U6‐DR_CMV‐minidCas13X.1‐REPAIRv1‐BGHpA_CMV‐EGFP‐BGHpA (mxABEv1), and U6‐DR_CMV‐minidCas13X.1‐REPAIRv2‐BGHpA_CMV‐EGFP‐BGHpA (mxABEv2, Addgene, #171383) were gifted by professor Yang Hui from Shanghai Research Center for Brain Science and Brain‐Inspired Intelligence, Chinese Academy of Sciences, Shanghai, China [10]. For target site gRNAs clone, briefly, oligos were synthesized and then annealed in a Bio‐Rad thermocycler. Then, the annealed fragment was ligated into BpiI (Thermo Scientific, FD1014)‐digested mxABEv1 or mxABEv2 backbones using T4 DNA ligase (Thermo Scientific, EK0031). All primers used in this study are provided in Table S1.

### 4.2 Cell Culture, Transfection, and Flow Cytometry Analysis

The HEK293T cell line was cultured in Dulbecco's modified Eagle's medium (Gibco, C11995500BT) supplemented with 10% fetal bovine serum (Vivacell, C04001) and 1% Pen‐Strep‐Glutamine (Vivacell, C3420‐0100) in a humidified incubator at 37°C with 5% CO_2_. Transfection of HEK293T cells was conducted with Lipofectamine 3000 (Invitrogen, L3000015) following the manufacturer's manual.

### 4.3 Sanger Sequencing, Deep Sequencing, and RNA‐seq Analysis

For Sanger and deep sequencing, HEK293T cells were cultured in 12‐well plates with 70% confluence, and 24 h later they were transfected by 3 µg of mxABEv1 or mxABEv2 plasmids with or without different sgRNA with Lipofectamine 3000. After 48 h, about 40,000 fluorescence‐positive cells were sorted by a MoFlo Astrios EQ flow cytometer machine (Beckman Coulter, USA). The total RNA of sorted cells was extracted from cells using RNA Isolation Reagent (Qiagen, 74034) and then converted to cDNA using a reverse transcription kit (Promega, A2801) following the manufacturer's manual. The target region for Sanger sequencing was amplified from cDNA with KOD Super‐Fidelity DNA Polymerase (TOYOBO, KOD‐401). The Sanger sequencing was performed by BGI Liuhe (Beijing) Technology Co., Ltd. and the A‐to‐G (I) editing rate was quantified by EditR [22], while deep sequencing was performed by Azenta Life Sciences (Suzhou). The libraries were prepared with the Nextera XT DNA Library Prep Kit following the standard manufacturer manual and sequenced on a NovaSeq. Sequencing data were first demultiplexed by Cutadapt (v.2.8) based on sample barcodes. The demultiplexed reads were then processed by CRISPResso2 for the quantification of A‐to‐I conversion efficiency at target site [23].

For RNA‐seq analysis, HEK293T cells were cultured in 10‐cm dishes with 70% confluence and transfected with 35 µg of plasmids to express editor with Lipofectamine 3000. About 500,000 fluorescence‐positive cells were collected by FACS, and RNA was extracted and then reverse‐transcribed (RT) to cDNA, which was used for transcriptome‐wide RNA‐seq. An average of the three repeats is presented. The mRNA sequencing (high throughput) was performed using Illumina Genome Analyzer at Tiangen Biotech Co. Ltd., and the adapters were removed using Trimmomatic (v0.36) during sequencing. RNA‐seq reads were aligned to the hg38 reference genome with Hisat2 (v.2.0.4). RNA editing sites were calculated using REDItools with the following parameters: ‐t 24 ‐e ‐d ‐l ‐U [AG or TC or CT or GA] ‐p ‐u ‐m20 ‐T6‐0 ‐W ‐v 1 ‐n 0.0. To identify single‐nucleotide variants, reads were processed using SplitNCigarReads in the GATK toolkit, and variants were called with HaplotypeCaller using the dbSNP database. To filter out known SNP loci, the dbSNP (v.151) database was downloaded from the University of Washington EVS website (https://evs.gs.washington.edu/EVS/), and overlapping sites were removed by matching chromosome and genomic coordinate. Sites supported by fewer than 20 reads were further filtered. Finally, sites overlapping the union of sites detected in the HEK293T no‐plasmid‐transfection group were removed. All cited databases are publicly available and fully accessible without access restrictions.

### 4.4 Immunofluorescence

Cells were fixed with 4% paraformaldehyde at room temperature for 15 min, washed in PBS, and incubated with 0.3% Triton X‐100 buffer (Solarbio, P1080) for 10 min. Then, the samples were blocked in PBS containing 10% donkey serum (Origene, ZLI‐9056) at 37°C for 1 h and incubated overnight with the primary antibody at 4°C. After PBS washes, they were incubated with the corresponding secondary antibody conjugated with fluorescence at room temperature for 1 h. Nuclei were incubated with DAPI staining solution (Solarbio, ID2250) for 10 min at room temperature in the dark. Images were acquired with a Leica STELLARIS 5 confocal laser scanning microscope. All antibodies used in this study are provided in Table S2.

### 4.5 qPCR

Total mRNA was extracted using RNA Isolation Reagent (Qiagen, 74034) and converted with a reverse transcription kit (Promega, A2801) following the manufacturer's manual. Quantitative PCR (qPCR) was performed with the cDNA for each sample on a Bio‐Rad CFX real‐time system using SYBR qPCR Master Mix (Promega, A6002). Relative expression was calculated by 2^(−ΔΔCt)^ with GAPDH as reference. And target genes were normalized by that of control groups [24]. All primers are provided in Table S1.

### 4.6 Western Blot Analysis

Each 2×10^6^ cells were lysed with 100 µL RIPA buffer supplemented with protease and phosphatase inhibitor (BestBio, BB‐3321). Protein concentration was determined with an Enhanced BCA Protein Assay Kit (Beyotime, P0010). Samples were mixed with 5×SDS‐PAGE protein loading buffer and boiled at 100°C for 10 min. Note that 30 µg protein per lane was loaded into 4%–20% SurePage gels (GenScript, M00655) and transferred to a PVDF membrane using Trans‐Blot Turbo Transfer System (Bio‐Rad). The membranes were blocked in 5% nonfat milk in TBST buffer, followed by incubating with the specific primary antibody at 4°C overnight and with the secondary antibody specific to the IgG of the species of primary antibody at room temperature for 1 h. Images were acquired with Odyssey CLx Infrared Imaging System (LI‐COR, USA) and quantified with ImageJ. Antibodies are listed in Table S2.

### 4.7 Wnt/β‐Catenin Activation Assay

To evaluate activation of the Wnt signaling pathway, HEK293T cells were seeded in 96‐well plates (10,000 cells per well). After 24 h, cells were transfected with 100 ng of mxABEv1 plasmid with target (T41A) or nontarget gRNA (NT), or with empty vector (NC), together with 100 ng of TOPFlash (Beyotime, D2506) or FOPFlash (Beyotime, D2508) and 100 ng of Renilla luciferase expressing plasmid (Beyotime, D2762). After 48 h, the Dual‐Glo Luciferase Assay System (Beyotime, RG089) was used according to the manufacturer's protocol. The TOPFlash reporter provides a metric of β‐catenin activation when compared to the background as measured by the FOPFlash reporter under the same condition. The accumulated β‐catenin activated the TOPFlash reporter by binding the promoter region but not the FOPFlash reporter, which contains a mutation on the promoter region. Folding activation was calculated as (TOPFlash/Renilla) ÷ (FOPFlash/Renilla) for each well, and all values were then normalized to the mean of the NT group (set to 1).

### 4.8 Cell Growth

The culture‐insert (Ibidi, 80209) was placed on a 12‐well plate, and 5000 HEK293T cells were seeded in each of the two chambers of the culture‐insert that prevent cell growth in the center of the well. After 24 h, cells were transfected with plasmids using lipofectamine 3000, as described above and incubated. Note that 24 h post‐transfection, the culture‐inserts were removed and created a cell‐free gap to allow for cell growth toward the center of the well. Phase‐contrast images of the identical field were taken at 0 h and 24 h after insert removal. The uncovered central area was measured with ImageJ and growth area was calculated as: (central gap area at 0 h) − (central gap area at 24 h).

### 4.9 Animal Studies

We conducted all animal care and experimentation in accordance with the Association for Assessment and Accreditation of Laboratory Animal Care guidelines and with approval from the University of Army Medical Animal Care and Use Committees. Mice were housed for 12‐h dark/light cycles with free access to food and water in SPF feeding environment before intervention. Sftpc‐CreERT2 transgenic mice (homozygous) (purchased from Jackson Laboratory, catalog number: 028054) and Rosa26‐RFP transgenic mice (homozygous) (presented by Shanghai Institute of Biological Sciences, Chinese Academy of Sciences) were bred and identified Sftpc‐CreERT2.

For in vivo delivery of LPS, mice were treated with LPS (10 mg/kg) intranasally (Sigma, L4005). The LPS solution was administered alternately in both nostrils.

For CT scanning, mice were anesthetized with an intraperitoneal injection of Pentobarbital (60 mg/kg) and then scanned with a CT system (Philips Brilliance ict, Netherlands). The scanning parameters were as follows: 120 kV, 150 mAs, 0.25 pitch, 0.4 s rotation time. Lung injury was quantified by measuring the mean Hounsfield Units (HU) from micro‐CT images using the Philips DICOM Viewer 3.0 L1 SP15 software. After adjusting the images to the LUNG window, the free selection tool was used to calculate the average HU across the entire lung lobe. Higher HU values indicate greater consolidation and/or oedema, corresponding to more severe lung injury.

For histology analysis, the lung tissues were cut coronally to obtain 5 µm and were stained with Hematoxylin and Eosin Staining Kit (Beyotime, C0105). Lung injury scoring was done as previously described [25].

### 4.10 AAV6.2FF Production and Delivery

AAV6.2FF serotype was used in this study. The mxABEv1 plasmid with target or nontarget gRNA was packaged into AAV6.2FF vehicle, and the AAV vectors were packaged by OBiO Technology, Shanghai, China. Vector genomes were quantified by TaqMan qPCR assay by targeting the inverted terminal repeat sequence of the AAV genome using the primers Forward: 5′‐TTACGCTATGTGGATACGC‐3′; Reverse: 5′‐AGAGACAGCAACCAGGAT‐3′. Endotoxin levels were assessed using the Gel Clot Limulus Amebocyte Lysate (LAL) assay and were confirmed to be less than 10 EU/mL. The ratio of empty‐to‐full capsids was determined via transmission electron microscopy (TEM), revealing a high packaging efficiency with less than 2.5% empty capsids (ranging from 1.09% to 2.3%). Mice were intratracheally administered 40 µL 10^11^ v.g. of an AAV6.2FF vector through air pump needle under the assistance of small animal laryngoscope.

### 4.11 In Vivo RNA‐Editing Analysis

Mice were euthanized and the lungs were collected and processed into a single‐cell suspension. And the cells were permeabilized for 1 h and stained by FITC‐conjugated monoclonal mouse HA‐Tag antibody (GenScript, A01621, 1:1000) for 1 h. FITC^+^ cells were sorted by a MoFlo Astrios EQ flow cytometer machine (Beckman Coulter, USA). The total RNA was extracted and converted to cDNA as described previously. The target region for Sanger sequencing was amplified with KOD Super‐Fidelity DNA Polymerase (TOYOBO, KOD‐401). The Sanger sequencing was performed by BGI Liuhe (Beijing) Technology Co., Ltd. and the A‐to‐I editing rate was quantified by EditR [22].

### 4.12 In Vivo off‐Target Analysis

On day 14, HA^+^ lung cells of treated mice were sorted. High‐throughput mRNA sequencing was performed by Tiangen Biotech Co. Ltd. using the Illumina Genome Analyzer platform. Adapter sequences were trimmed with Trimmomatic (v0.39) during preprocessing. RNA‐seq reads were aligned to the GRCm39 reference genome using Hisat2 (v2.2.1). RNA editing site detection was carried out with the GATK (v4.1.5.0). Variant calling was performed with HaplotypeCaller, applying a minimum confidence threshold of 20. Subsequent filtering with VariantFiltration was used to exclude variants with FS > 30.0, QD < 2.0, or DP < 20. The union of variants identified in the NC group was used as the baseline to remove overlapping sites, and variants located within exonic regions were also excluded.

### 4.13 Blood Gas Analysis

Mice were anesthetized and disinfected with 70% ethanol, and the carotid artery was exposed by blunt dissection. Approximately 100 µL of arterial blood was collected using a heparinized arterial blood gas syringe and immediately loaded into a CG4+ Blood Gas Biochemistry Cartridge and analyzed on an i‐STAT Analyzer (Abbott Point of Care Inc., model 300‐G) according to the manufacturer's instructions.

### 4.14 Lung Function Analysis

Pulmonary function was evaluated using an unrestrained whole‐body plethysmography system (WBP‐MR, Tawang Technology, China). The system was standardized and calibrated before each experiment. Mice were placed individually in the chamber for a 20‐min recording session, and respiratory parameters were continuously monitored with a sampling interval of 5 s. Between animals, the chamber was disinfected with 70% ethanol and aerated for at least 10 min to eliminate residual odor. The last 5 min of each recording was used for quantitative analysis. Parameters assessed included PEF, expiratory flow at 50% of tidal volume (EF50), and MV.

### 4.15 Statistical Analysis

All statistical values were presented as mean ± SEM. Normality and homogeneity of variance were assessed using the Shapiro–Wilk test and the F‐test or Brown–Forsythe test, respectively. For comparisons between two groups, an unpaired two‐tailed Student's *t*‐test was used when assumptions were met. Welch's *t*‐test was applied when variances were unequal, and the Mann–Whitney *U* test was used for non‐normally distributed data. For multiple group comparisons, ordinary one‐way ANOVA followed by Tukey's post hoc test was performed. When variances were unequal, the Brown‐Forsythe–Welch ANOVA with Dunnett's T3 multiple comparisons test was utilized. Multi‐factor comparisons were analyzed using two‐way ANOVA followed by Bonferroni's post hoc test for pairwise comparisons. *p* < 0.05 was considered statistically significant. GraphPad Prism 10.0 (GraphPad Software, San Diego, CA, USA) was used for statistical analysis.

## Author Contributions

W.L. and W.B. were the main researchers of this study. S.H., J.D., H.Z., D.W., Q.C., and C.G. performed the technical work. L.Z. and A.Z. performed supervision and data curation. P.L., M.W., L.L., and J.J. planned the study, wrote the manuscript. All authors read and approved the final manuscript.

## Funding

This work was supported by the National Natural Science Foundation of China (82020108021, U23A20393, 82272908, and 32470022), The Outstanding Young Talents of National Defense Biotechnology (2023‐JCJQ‐ZQ‐001), the Outstanding Youth Science Fund of Chongqing Municipal Science and Technology Bureau (CSTB2023NSCQ‐JQX0033); Foundation of State Key Laboratory of Trauma and Chemical Poisoning (SKLKF202211 and SKLO202406), and Noncommunicable Chronic Diseases‐National Science and Technology Major Project of China (2024ZD0541500).

## Ethics Statement

Ethics approval for this study was obtained from the Laboratory Animal Welfare and Ethics Committee of Third Military Medical University (No. AMUWEC20201178).

## Conflicts of Interest

The authors declare no conflicts of interest.

## Supporting information




**Supporting File 1**: mco270716‐sup‐0001‐SuppMat.docx.

## Data Availability

All raw sequencing data generated during this study have been deposited in the Genome Sequence Archive (GSA) at the National Genomics Data Center (NGDC), China National Center for Bioinformation/Beijing Institute of Genomics, Chinese Academy of Sciences. The RNA‐seq data for 293T cells are available under accession number HRA015823, and the in vivo off‐target sequencing data are available under accession number CRA037227. These data can be accessed at https://ngdc.cncb.ac.cn/.

## References

[1] G. Bellani , J. G. Laffey , T. Pham , et al., “Epidemiology, Patterns of Care, and Mortality for Patients With Acute Respiratory Distress Syndrome in Intensive Care Units in 50 Countries,” JAMA 315, no. 8 (2016): 788–800. 26903337 10.1001/jama.2016.0291

[2] M. C. Basil , J. Katzen , A. E. Engler , et al., “The Cellular and Physiological Basis for Lung Repair and Regeneration: Past, Present, and Future,” Cell Stem Cell 26, no. 4 (2020): 482–502. 32243808 10.1016/j.stem.2020.03.009 PMC7128675

[3] N. J. Meyer , L. Gattinoni , and C. S. Calfee , “Acute Respiratory Distress Syndrome,” Lancet 398, no. 10300 (2021): 622–637. 34217425 10.1016/S0140-6736(21)00439-6 PMC8248927

[4] C. E. Barkauskas , M. J. Cronce , C. R. Rackley , et al., “Type 2 Alveolar Cells Are Stem Cells in Adult Lung,” Journal of Clinical Investigation 123, no. 7 (2013): 3025–3036. 23921127 10.1172/JCI68782 PMC3696553

[5] W. J. Zacharias , D. B. Frank , J. A. Zepp , et al., “Regeneration of the Lung Alveolus by an Evolutionarily Conserved Epithelial Progenitor,” Nature 555, no. 7695 (2018): 251–255. 29489752 10.1038/nature25786 PMC6020060

[6] R. L. Zemans , N. Briones , M. Campbell , et al., “Neutrophil Transmigration Triggers Repair of the Lung Epithelium via Beta‐Catenin Signaling,” PNAS 108, no. 38 (2011): 15990–15995. 21880956 10.1073/pnas.1110144108 PMC3179042

[7] A. N. Nabhan , D. G. Brownfield , P. B. Harbury , M. A. Krasnow , and T. J. Desai , “Single‐Cell Wnt Signaling Niches Maintain Stemness of Alveolar Type 2 Cells,” Science 359, no. 6380 (2018): 1118–1123. 29420258 10.1126/science.aam6603 PMC5997265

[8] O. O. Abudayyeh , J. S. Gootenberg , B. Franklin , et al., “A Cytosine Deaminase for Programmable Single‐Base RNA Editing,” Science 365, no. 6451 (2019): 382–386. 31296651 10.1126/science.aax7063 PMC6956565

[9] O. O. Abudayyeh , J. S. Gootenberg , S. Konermann , et al., “C2c2 is a Single‐Component Programmable RNA‐Guided RNA‐Targeting CRISPR Effector,” Science 353, no. 6299 (2016): aaf5573. 27256883 10.1126/science.aaf5573 PMC5127784

[10] C. Xu , Y. Zhou , Q. Xiao , et al., “Programmable RNA Editing With Compact CRISPR‐Cas13 Systems From Uncultivated Microbes,” Nature Methods 18, no. 5 (2021): 499–506. 33941935 10.1038/s41592-021-01124-4

[11] B. L. Ellis , M. L. Hirsch , J. C. Barker , and J. P. Connelly , “Steininger RJ 3rd, Porteus MH. A Survey of Ex Vivo/in Vitro Transduction Efficiency of Mammalian Primary Cells and Cell Lines With Nine Natural Adeno‐associated Virus (AAV1‐9) and One Engineered Adeno‐Associated Virus Serotype,” Virology Journal 10 (2013): 74. 23497173 10.1186/1743-422X-10-74 PMC3607841

[12] M. P. Seiler , A. D. Miller , J. Zabner , and C. L. Halbert , “Adeno‐Associated Virus Types 5 and 6 Use Distinct Receptors for Cell Entry,” Human Gene Therapy 17, no. 1 (2006): 10–19. 16409121 10.1089/hum.2006.17.10

[13] M. P. Limberis , L. H. Vandenberghe , L. Zhang , R. J. Pickles , and J. M. Wilson , “Transduction Efficiencies of Novel AAV Vectors in Mouse Airway Epithelium in Vivo and Human Ciliated Airway Epithelium in Vitro,” Molecular Therapy 17, no. 2 (2009): 294–301. 19066597 10.1038/mt.2008.261 PMC2835069

[14] M. H. Kang , L. P. van Lieshout , L. Xu , et al., “A Lung Tropic AAV Vector Improves Survival in a Mouse Model of Surfactant B Deficiency,” Nature Communications 11, no. 1 (2020): 3929. 10.1038/s41467-020-17577-8 PMC7414154 32764559

[15] Q. Xiao , Z. Xu , Y. Xue , et al., “Rescue of Autosomal Dominant Hearing Loss by in Vivo Delivery of Mini dCas13X‐Derived RNA Base Editor,” Science Translational Medicine 14, no. 654 (2022): eabn0449. 35857824 10.1126/scitranslmed.abn0449

[16] H. Clevers , K. M. Loh , and R. Nusse , “Stem Cell Signaling. An Integral Program for Tissue Renewal and Regeneration: Wnt Signaling and Stem Cell Control,” Science 346, no. 6205 (2014): 1248012. 25278615 10.1126/science.1248012

[17] A. N. Nabhan , J. D. Webster , J. J. Adams , et al., “Targeted Alveolar Regeneration With Frizzled‐Specific Agonists,” Cell 186, no. 14 (2023): 2995–3012.e15. 37321220 10.1016/j.cell.2023.05.022

[18] H. Chen , C. Lu , S. J. Lee , and Y. Li , “Protocol to Generate and Characterize Potent and Selective WNT Mimetic Molecules,” STAR Protocol 1, no. 1 (2020): 100043. 10.1016/j.xpro.2020.100043 PMC7580111 33111090

[19] M. Kahn , “Can We Safely Target the WNT Pathway,” Nature Reviews Drug Discovery 13, no. 7 (2014): 513–532. 24981364 10.1038/nrd4233 PMC4426976

[20] G. Li , M. Jin , Z. Li , et al., “Mini‐dCas13X‐Mediated RNA Editing Restores Dystrophin Expression in a Humanized Mouse Model of Duchenne Muscular Dystrophy,” Journal of Clinical Investigation 133, no. 3 (2023): e162809. 36512423 10.1172/JCI162809 PMC9888377

[21] S. Shen , P. Wang , P. Wu , et al., “CasRx‐Based Wnt Activation Promotes Alveolar Regeneration While Ameliorating Pulmonary Fibrosis in a Mouse Model of Lung Injury,” Molecular Therapy: The Journal of the American Society of Gene Therapy 32, no. 11 (2024): 3974–3989. 39245939 10.1016/j.ymthe.2024.09.008 PMC11573616

[22] M. G. Kluesner , D. A. Nedveck , W. S. Lahr , et al., “EditR: A Method to Quantify Base Editing From Sanger Sequencing,” CRISPR Journal 1, no. 3 (2018): 239–250. 31021262 10.1089/crispr.2018.0014 PMC6694769

[23] K. Clement , H. Rees , M. C. Canver , et al., “CRISPResso2 Provides Accurate and Rapid Genome Editing Sequence Analysis,” Nature Biotechnology 37, no. 3 (2019): 224–226. 10.1038/s41587-019-0032-3 PMC6533916 30809026

[24] K. J. Livak and T. D. Schmittgen , “Analysis of Relative Gene Expression Data Using Real‐Time Quantitative PCR and the 2(‐Delta Delta C(T)) Method,” Methods 25, no. 4 (2001): 402–408. 11846609 10.1006/meth.2001.1262

[25] G. Matute‐Bello , G. Downey , B. B. Moore , et al., “An Official American Thoracic Society Workshop Report: Features and Measurements of Experimental Acute Lung Injury in Animals,” American Journal of Respiratory Cell and Molecular Biology 44, no. 5 (2011): 725–738. 21531958 10.1165/rcmb.2009-0210ST PMC7328339

