# Supplementary material for: Mini‐Catalytically Inactive Cas13X‐Derived RNA Base Editing of β‐Catenin Attenuates Pulmonary Damage in a Murine Acute Lung Injury Model
Source: MedComm (2020). 2026 Mar 30;7(4):e70716. doi: 10.1002/mco2.70716 (PMC13042493; doi:10.1002/mco2.70716)
Supplement: Supplementary file 1 — Supporting File 1: mco270716‐sup‐0001‐SuppMat.docx. [file MCO2-7-e70716-s001.docx]

**Mini-catalytically inactive Cas13X-derived RNA base editing of β-catenin** **attenuates pulmonary damage in a murine acute lung injury model**

Wenyi Liu,^1,5^ Wanda Bi,^1,5^ Saiying Hou,^1^ Juan Du,^1^ Ling Zeng,^1^ Anqiang Zhang,^1^ Huacai Zhang,^1^ Dalin Wen,^1^ Qingli Cai,^1^ Chu Gao,^1^ Ping Lin,^2*^ Min Wu,^3*^ Li Li^1,4,*^，Jianxin Jiang^1*^

^1^Department of Trauma Medical Center, Daping Hospital, State Key Laboratory of Trauma and Chemical Poisoning, Army Medical University, Chongqing, 400042, China.

^2^Biological Science Research Center, Southwest University, Chongqing, 400716, China

^3^Wenzhou Institute, University of Chinese Academy of Sciences, Wenzhou, Zhejiang 325000, China

^4^Department of Respiratory Medicine, Daping Hospital, Third Military Medical University (Army Medical University), Chongqing, 400042, China

^5^These authors contributed equally

^*^Correspondence: Jianxin Jiang, [hellojjx@tmmu.edu.cn](mailto:hellojjx@tmmu.edu.cn), Department of Trauma Medical Center, Daping Hospital, State Key Laboratory of Trauma and Chemical Poisoning, Army Medical University, Chongqing, 400042, China, or Li Li, [dpyyhxlili@tmmu.edu.cn](mailto:dpyyhxlili@tmmu.edu.cn), or Min Wu, [minwoo2022@126.com](mailto:minwoo2022@126.com), or Ping Lin, [linpingswu@swu.edu.cn](mailto:linpingswu@swu.edu.cn)

Lead Contact: Jianxin Jiang, [hellojjx@tmmu.edu.cn](mailto:hellojjx@tmmu.edu.cn)

**Supplementary materials**

**Table S1.** All primers used in this study.

| **experiment** | **Name** | **sequence (5'-3')** |
| --- | --- | --- |
| sgRNA for human CTNNB1 | sg-hCTNNB1-A25-F | ACCGTTACCACTCAGAGAAGGAGCTGTGGCAGTGGCACCAGAATGGATTCCAGA |
|  | sg-hCTNNB1-A25-R | CAGCTCTGGAATCCATTCTGGTGCCACTGCCACAGCTCCTTCTCTGAGTGGTAA |
|  | sg-hCTNNB1-A26-F | ACCGTACCACTCAGAGAAGGAGCTGTGGCAGTGGCACCAGAATGGATTCCAGAG |
|  | sg-hCTNNB1-A26-R | CAGCCTCTGGAATCCATTCTGGTGCCACTGCCACAGCTCCTTCTCTGAGTGGTA |
|  | sg-hCTNNB1-A27-F | ACCGACCACTCAGAGAAGGAGCTGTGGCAGTGGCACCAGAATGGATTCCAGAGT |
|  | sg-hCTNNB1-A27-R | CAGCACTCTGGAATCCATTCTGGTGCCACTGCCACAGCTCCTTCTCTGAGTGGT |
|  | sg-hCTNNB1-A28-F | ACCGCCACTCAGAGAAGGAGCTGTGGCAGTGGCACCAGAATGGATTCCAGAGTC |
|  | sg-hCTNNB1-A28-R | CAGCGACTCTGGAATCCATTCTGGTGCCACTGCCACAGCTCCTTCTCTGAGTGG |
|  | sg-hCTNNB1-A29-F | ACCGCACTCAGAGAAGGAGCTGTGGCAGTGGCACCAGAATGGATTCCAGAGTCC |
|  | sg-hCTNNB1-A29-R | CAGCGGACTCTGGAATCCATTCTGGTGCCACTGCCACAGCTCCTTCTCTGAGTG |
|  | sg-hCTNNB1-A30-F | ACCGACTCAGAGAAGGAGCTGTGGCAGTGGCACCAGAATGGATTCCAGAGTCCA |
|  | sg-hCTNNB1-A30-R | CAGCTGGACTCTGGAATCCATTCTGGTGCCACTGCCACAGCTCCTTCTCTGAGT |
| sgRNA for mouse CTNNB1 | sg-mCTNNB1-A27-F | ACAGGCCACTCAGGGAAGGAGCTGTGGCGGTGGCACCAGAATGGATTCCAGAAT |
|  | sg-mCTNNB1-A27-R | CAGCATTCTGGAATCCATTCTGGTGCCACCGCCACAGCTCCTTCCCTGAGTGGC |
| PCR primer | hCTNNB1-F | TTTGATGGAGTTGGACA |
|  | hCTNNB1-R | TCTCAGGGAACATAGC |
|  | mCTNNB1-F | CTACTCAAGCTGACCTG |
|  | mCTNNB1-R | CAAACTGCGTGGATG |

**Table S2.** All antibodies used in this study.

| **Antibody** | **Product Code** | **Manufacturer** | **Species** | **Dilution** | **Application** |
| --- | --- | --- | --- | --- | --- |
| β-catenin antibody | ab22656 | Abcam | mouse | 1:1000 | WB |
| phosphorylated β-catenin antibody | CST9561 | CST | rabbit | 1:1000 | WB |
| β-actin antibody | 20536-1-AP | Proteintech | mouse | 1:4000 | WB |
| Lamin B1 antibody | 66095-1-PBS | Proteintech | mouse | 1:3000 | WB |
| α-SMA antibody | 14395-1-AP | Proteintech | rabbit | 1:5000 | WB |
| COL1A1 antibody | BA0325 | Boster | rabbit | 1:1000 | WB |
| GAPDH antibody | 60004-1-Ig | Proteintech | mouse | 1:5000 | WB |
| 680RD Donkey anti-Rabbit antibody | 926-68073 | LI-COR | donkey | 1:10000 | WB |
| 800CW Goat anti-Mouse antibody | 926-32210 | LI-COR | goat | 1:10000 | WB |
| β-catenin antibody | ab32572 | Abcam | rabbit | 1:1000 | IF |
| HA-Tag antibody | A01621 | Genscript | mouse | 1:250 | IF |
| Sftpc antibody | ab40879 | Abcam | rabbit | 1:1000 | IF |
| Ki67 antibody | M00254-8 | Boster | mouse | 1:400 | IF |
| Fra1 antibody | PA576185 | Invitrogen | rabbit | 1:200 | IF |
| Ppard antibody | GTX79186 | Genetex | rabbit | 1:200 | IF |
| α-SMA antibody | 14395-1-AP | Proteintech | rabbit | 1:100 | IF |
| COL1A1 antibody | BA0325 | Boster | rabbit | 1:100 | IF |
| AF594, donkey anti-rabbit antibody | ab150064 | Abcam | donkey | 1:1000 | IF |
| AF488, goat anti-mouse antibody | ab150113 | Abcam | goat | 1:1000 | IF |
| AF647, goat anti-rabbit antibody | 4414 | CST | goat | 1:1000 | IF |


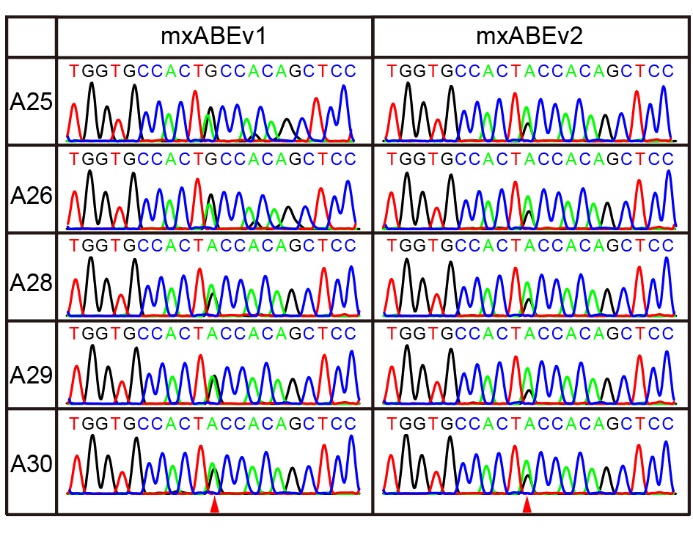


**Figure S1.** Sanger sequencing results for in vitro editing with base-editors of different gRNAs, with red arrowheads indicating the target nucleotide.


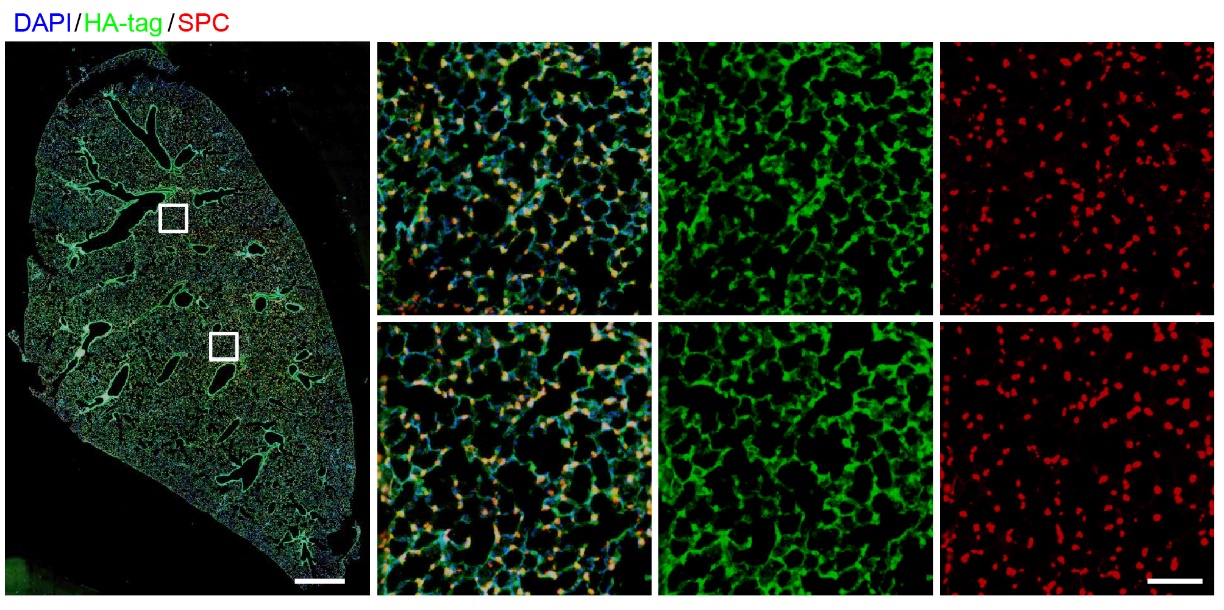


**Figure S2.** Representative immunofluorescence images of AAV-transduced (HA⁺, green) cells, AT2 cells (Sftpc⁺, red), and nuclei (DAPI^+^, blue) of mouse lungs 14 days following AAV-mxABE-NT (NT) intratracheal instillation. Scale bar, left=1mm, Right=50 μm.


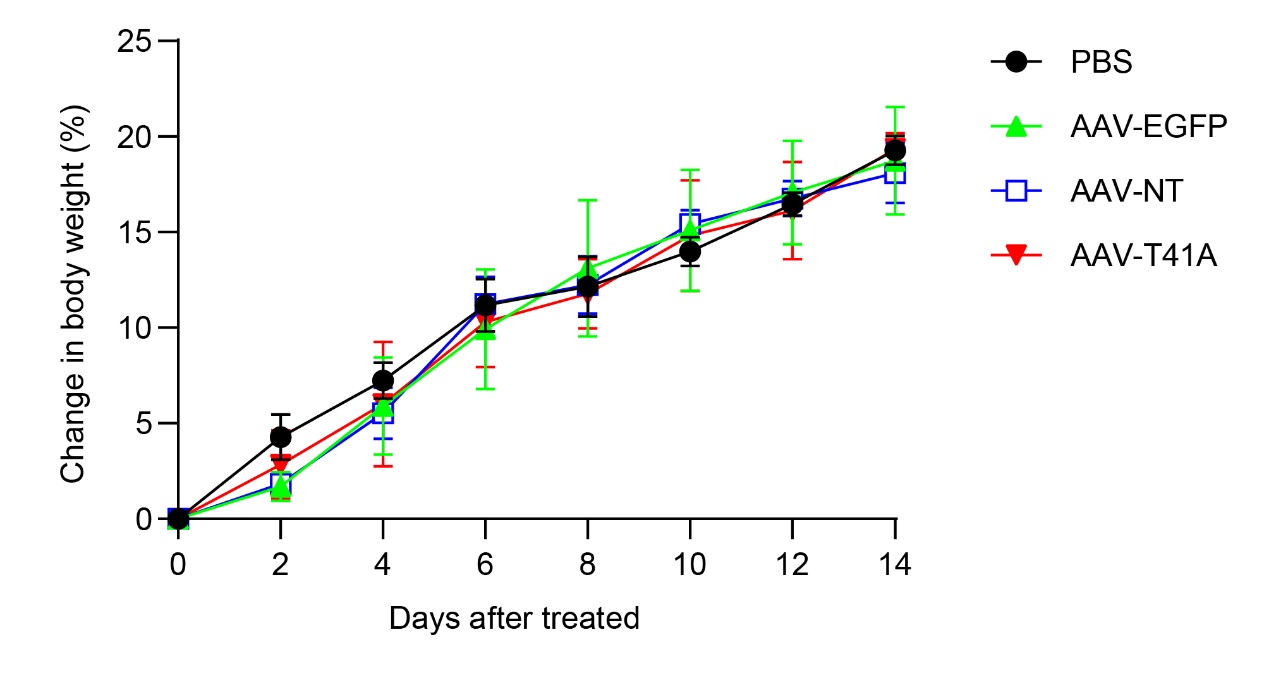
**Figure S3.** Mean weight measurements with SEM of mice treated with PBS (PBS), AAV6.2FF vector containing EGFP (AAV-EGFP), mxABEv1-NT (AAV-NT), or mxABEv1-T41A (AAV-T41A). n = 3 per group.


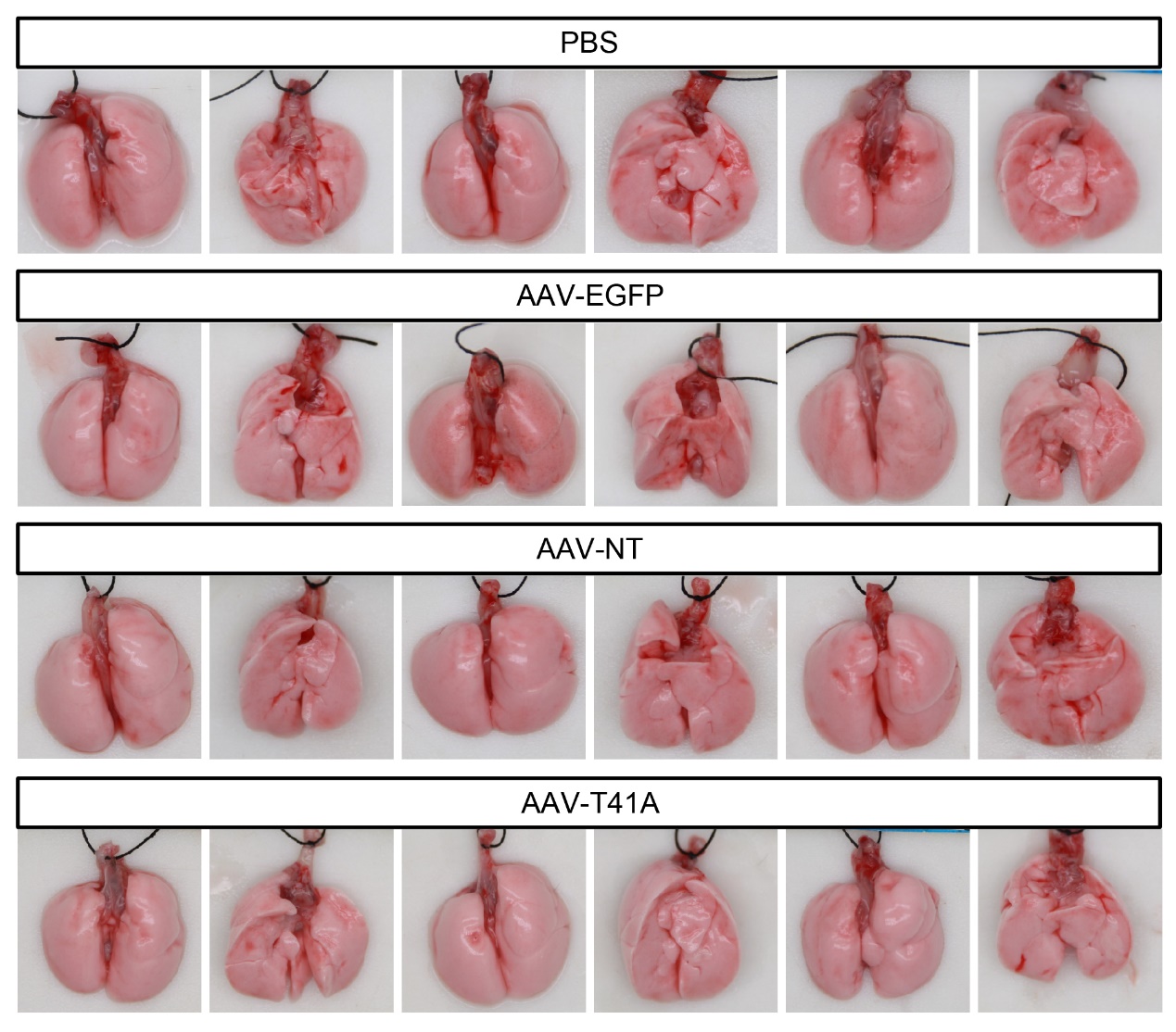
 **Figure S4.** Gross morphology of representative lungs 14d after intratracheal delivery of PBS, AAV6.2FF vector containing EGFP (AAV-EGFP), mxABEv1-NT (AAV-NT), or mxABEv1-T41A (AAV-T41A).
